# Chronic toxicity of dissolved barium and sodium chloride to the water flea *Ceriodaphnia dubia*: implications for unconventional gas flowback–produced waters

**DOI:** 10.1093/etojnl/vgae019

**Published:** 2025-01-06

**Authors:** Daniel J Willems, Anupama Kumar, Dayanthi Nugegoda

**Affiliations:** Ecotoxicology Research Group, School of Science, Royal Melbourne Institute of Technology (RMIT) University, Bundoora West Campus, Victoria, Australia; Environment, Commonwealth Scientific and Industrial Research Organisation (CSIRO), Waite Campus, Adelaide, South Australia, Australia; Environment, Commonwealth Scientific and Industrial Research Organisation (CSIRO), Waite Campus, Adelaide, South Australia, Australia; Ecotoxicology Research Group, School of Science, Royal Melbourne Institute of Technology (RMIT) University, Bundoora West Campus, Victoria, Australia

**Keywords:** barite, salinity, binary toxicity, sulfate, flowback produced water

## Abstract

Unconventional gas flowback–produced waters, particularly those of marine origin from shale gas, can contain elevated concentrations of barium (Ba) and sodium chloride (NaCl). There are limited Ba water quality guideline values to help assess the potential risk of Ba exposure to freshwater biota. Barium toxicity is heavily influenced by sulfate concentrations as Ba and sulfate react, forming the highly water-insoluble and thus less bioavailable Ba sulfate. To determine survival and reproductive impacts, the water flea *Ceriodaphnia dubia* was exposed to dissolved Ba, NaCl, and binary combinations of dissolved Ba and NaCl. No chronic lethal concentration (LC) endpoints could be determined for dissolved Ba-only exposure up to 16 mg/L due to near 100% parent survivorship across all treatments. The NaCl LC50 (95% credible intervals) = 708 (502–939) mg/L. The dissolved Ba reproductive effect concentration (EC) at EC20 was 0.95 (0.19–3.22) mg/L. Meanwhile, for NaCl, the EC10 and EC20 concentrations were 365 (149–651) mg/L and 510 (282–760) mg/L, respectively. The binary exposure of Ba and NaCl had limited meaningful data due to some experimental shortcomings (lack of Ba-only and NaCl-only controls). Despite this, at 410 mg/L NaCl, the dissolved Ba reproductive EC10 and EC20 were determined to be 8.87 [3.58–11.7] mg/L and 10.1 (5.64–11.8) mg/L, respectively. These results suggest that marginally increased NaCl concentrations alleviate Ba toxicity, particularly when Ba is at low concentrations. Further chronic studies are needed to account for Ba toxicity in dissolved and precipitated forms and derive a Ba guideline value for freshwater biota.

## Graphical Abstract

**Figure:**
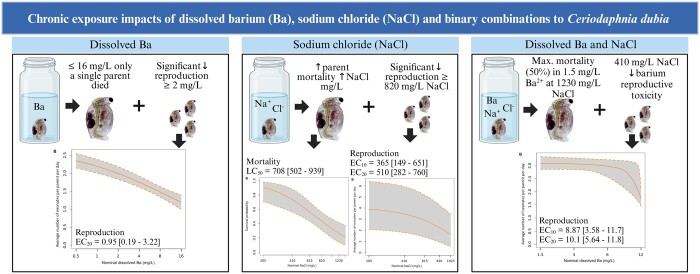

## Introduction

Onshore unconventional gas extraction utilizes hydraulic fracturing (fracking), which produces large volumes of waste waters per well (1.7–14.3 × 10^6^ L, Kondash et al., 2017) either as “flowback waters” (FWs) or produced waters (PWs). Flowback water is the initial return of hydraulic fracturing fluid, whereas PW is extracted from the formation throughout the remaining life of a well (Blewett et al., 2017). Accumulation and mixing of FW and PW occur (e.g., in storage tanks or holding ponds), and this water is commonly referred to as flowback-produced water (FPW; Blewett et al., 2017). These waters are chemically complex with many characterized chemicals (Elsner & Hoelzer, 2016; Ferrer & Thurman, 2015; National Industrial Chemicals Notification and Assessment Scheme, 2017; Stringfellow et al., 2014; Waxman et al., 2011) and uncharacterized chemicals, in addition to being temporally heterogeneous. Shale gas (SG) FPW typically has elevated salinity and inorganics compared with coal-bed methane (CBM) FPW (Silva et al., 2017; Willems et al., 2022). The salinity of FPW is a common source of toxicity to varying degrees in freshwater organisms (Folkerts et al., 2020; Golding et al., 2022; Willems et al., 2022).

Flowback-produced water can enter and contaminate surface waters through spills, leaks, discharges, or run-off events (Folkerts et al., 2020; Willems et al., 2022, 2023), creating potential toxicity to freshwater biota. The toxicity of FPW in surface water environments is heavily influenced by the volume of FPW and the chemistry of the receiving water body and its associated flow rates (McLaughlin et al., 2020; Ni et al., 2022). Spills and leaks commonly involve untreated FPW due to their accidental or unplanned nature, posing a greater risk to freshwater biota. Discharges of FPW, like those permitted under the National Pollutant Discharge Elimination System (NPDES) in the United States, require treatment before discharge (United States Environmental Protection Agency [USEPA], 2010). Spills or leaks of FPW are not a rare occurrence. From 2005 to 2012, in Alberta, Canada, > 2500 PW spills occurred, and 113 of these spills entered freshwater lakes or streams (Alessi et al., 2017; Folkerts et al., 2020; Goss et al., 2015). Data from 2005 to 2014 in the United States from > 30,000 wells across several states showed that 50% of spills were associated either with storage or transport of FPW (Patterson et al., 2017), with an estimate of 2% to 16% of wells reporting spills annually, with the largest volume spill reported at 3756 m^3^ (Patterson et al., 2017).

Barium (Ba) is frequently detected at considerable concentrations in FPW; Ba in SG can average 815 mg/L, whereas Ba in CBM averages 25 mg/L (Willems et al., 2022). Barium in FPW has multiple sources, including Ba sulfate (BaSO_4_, barite) as a weighing agent in hydraulic fracturing fluids/muds (Neff, 2008). Another source is the basin’s geology and the geogenic waters (Renock et al., 2016). Using scale inhibitors to prevent well infrastructure damage reduces dissolved sulfate concentrations in returning waters (Neff et al., 2011). Barium toxicity is strongly influenced by its solubility when complexed with certain anions. For example, the solubility of BaSO_4_ in water at 20°C is only 0.0025 g/L, and BaCl_2_ is 370 g/L in water at 25°C (Aylward & Findlay, 1974; Golding et al., 2018).

Sodium (Na^+^) and chloride (Cl^-^) are commonly the major ions with the highest concentrations in FPW. Particularly those from SG at 22,600 and 56,000 mg/L, respectively, whereas those from CBM are at 1,900 and 2,500 mg/L concentrations, respectively (Willems et al., 2022). This poses a toxicity concern to freshwater organisms (Folkerts et al., 2019; Golding et al., 2022). Despite both ions being essential for life, they can become toxic to biota at concentrations that exceed physiological requirements and thus affect osmoregulation. Severe salinity alterations can result in mortality, with less severe (sublethal) alterations causing reduced reproductive fitness, altered tissue and organ function, reduced disease resistance, and stunted growth and development (Barton & Iwama, 1991; Pickering, 1990; Schreck et al., 2001). Synthetic marine salts (derived from marine waters) are commonly used to assess salinity toxicity to freshwater invertebrates (Dowse et al., 2017; Kefford et al., 2023) and better represent the ionic composition of inland saline waters of Australia. However, in this study, NaCl was used to exclude the sulfate ion (${\text{SO}}_{4}^{2-}$) present in synthetic marine salts (Atkinson & Bingman, 1997) as it would react with dissolved Ba, forming the water-insoluble BaSO_4_.

Major ion composition and its ratios also have a major influence on salinity toxicity for freshwater invertebrates (Erickson et al., 2017; Kunz et al., 2013; Mount et al., 2019, 2016, 1997), as further discussed in Cañedo-Argüelles et al. (2016, 2013). Our study is particularly relevant to waters dominated by Na^+^ and Cl^-^ ions, such as SG FPW (Willems et al., 2022).

The objective of this study was to use a cladoceran model organism, *Ceriodaphnia dubia*, to assess the chronic survival and reproductive toxicity of prominent chemicals found in FPW (dissolved Ba, Na^+^, and Cl^−^) using single and binary combination exposures. Concentrations of these chemicals used in the toxicity testing represented those within FPW, particularly those from SG. To the authors’ current knowledge, no published literature has investigated the chronic binary toxicity of dissolved Ba and NaCl without confounding influences of ${\text{SO}}_{4}^{2-}$ in water fleas like *C. dubia.*

## Materials and methods

### Test materials

Barium as BaCl_2_⋅2H_2_O (99.999%) trace metal basis (CAS no. 10326-27-9), NaCl American Chemical Society (ACS) reagent of ≥99.0% (CAS no. 7647-14-5), and other salts used for culture media preparation were also of ACS or 99.999% trace metal basis grade and purchased from Sigma-Aldrich Pty, Australia. Stock preparations involved a 0.025 M dissolved Ba in Milli-Q water (18 MΩ·cm, Milli-Q, Millipore). A 5 M NaCl solution in Milli-Q water was used to adjust the moderately hard water–chloride (MHW-Cl, described below) chloride control to 500 µS/cm and other nominal NaCl concentration solutions in MHW-Cl to within a ± 2% tolerance with the aid of a calibrated conductivity probe and were kept constantly aerated in new and Milli-Q water-rinsed 25 L plastic carboys that were covered.

Moderately hard water–chloride modified up to 500 µS/cm using the 5 M NaCl stock formed the control testing water and was based on standard MHW with compositions of 96, 60, 60, and 4 mg/L of NaHCO_3_, CaSO_4_⋅2H_2_O, MgSO_4_, and KCl respectively (ASTM International, 2014). The only modifications were that the sulfate-containing salts CaSO_4_⋅2H_2_O and MgSO_4_ were substituted with chloride salts CaCl_2_⋅2H_2_O (51.2 mg/L) and MgCl_2_⋅6H_2_O (101.3 mg/L) to avoid BaSO_4_ precipitation. The modified MHW-Cl was not used for *C. dubia* culturing (see *Test organism and culture conditions* section), where standard dilute mineral water was used. Glassware used throughout experimentation was first washed in a solution of laboratory detergent (Pyroneg), rinsed with distilled water, then soaked for 24 hr in 5% v/v of concentrated (70%) nitric acid (HNO_3_) in Milli-Q water, then triple-rinsed with Milli-Q water.

### Test organism and culture conditions

The water flea *C. dubia* used in this study was from an established on-site culture at the CSIRO Environment laboratory at Urrbrae, South Australia. Mass cultures were maintained at 60 individuals in 800 ml of dilute mineral water (DMW) prescribed by USEPA (2002a). 1.5 L (20% v/v) of Perrier mineral water was diluted with 6 L of Milli-Q water. 0.5 µl/ml (3750 µl) of vitamin B12 (20 mg/L stock), 0.5 µl/ml (3750 µl) of selenium as Na_2_SeO_3_ (4 mg Se/L stock) also added and aerated overnight before use. The resulting aerated DMW pH was 7.9–8.3 (USEPA, 2002a) because insufficient aeration of freshly prepared DMW can result in mass mortality of the *C. dubia* culture.

Three times per week (Monday, Wednesday, and Friday) at renewals, food was added to DMW and thoroughly mixed before transfer of *C. dubia*. The primary food source was from a green microalgae *Raphidocelis subcapitata* concentrate fed at 2 × 10^5^ cells/mL, 1 µl/ml supernatant Algotene red marine phytoplankton *Dunaliella salina* (from a 12.5 g/L stock), and 1 µl/ml of supernatant from a 5.6 g/100 ml stock of finely grounded Tetramin tropical fish flakes, with ground particulates allowed to settle overnight before acquiring supernatant. All food/supplement stocks were prepared with Milli-Q and refrigerated at 4°C. Fish food stocks were prepared fresh weekly. Mass cultures were not actively aerated (USEPA, 2002a) and were incubated at 25°C ± 1°C with a 16:8-hr light:dark regimen using fluorescent tube lighting. *Raphidocelis subcapitata* was axenically cultured in A-MS media (Keating, 1985) under fluorescent lighting. The feeding/supplement concentrations used for culturing were maintained during toxicity testing, except that feeding was performed daily (every 24 hr) as required (USEPA, 2002b) during chronic toxicity testing (see *Chronic toxicity tests* section).

### Chronic toxicity tests

The chronic/reproductive tests were performed as guided by the USEPA (2002b) method for *C. dubia.* Tests were deemed valid and terminated on meeting the test criteria of 60% or more of surviving control females having three broods of offspring and reproducing ≥ 15 neonates in those three broods by the end of Day 8 of exposure (USEPA, 2002b). Tests were valid and terminated after Days 6 (NaCl only) or 7 (dissolved Ba only and binary dissolved Ba and NaCl). The control treatment was the modified MHW-Cl to 500 µS/cm in each of the three exposures. A single *C. dubia* neonate <24 hr of age and a third brood or greater from mass culture were placed into each 25 ml glass scintillation vial, with a 20 ml treatment volume. Each treatment solution was prepared daily with a slight excess for a whole treatment to ensure greater homogeneity and enable water sampling and water quality testing. Ten replicates per treatment were used. The feeding/supplement concentrations and incubator conditions (see *Test organism and culture conditions* section) matched those of culture conditions except for daily (24 hr) renewals during testing*. C. dubia* parent mortality was defined as complete immobilization of individuals, (i.e., when there was no visible swimming movement after a gentle swirl or agitation of solution). The number of neonates reproduced was recorded daily during renewals. Another set of matching 20 ml scintillation vials was used to assist in daily/24 hr renewals of *C. dubia* into fresh treatment solution with all conditions matching; the prior day set of vials was rinsed with some existing solution for each treatment to remove accumulated algae on the interior of the vial. Water samples were collected at test initiation 0 hr (new) and 24 hr (old) and repeated 24 hr prior (new) and at test completion (old) to evaluate the water quality parameters of temperature (°C), dissolved oxygen (DO, mg/L), pH, and electrical conductivity (µS/cm) using a Hach HQ40d water quality meter and probe set. At the same period, another subset of water was sampled to analytically measure Ba and sodium content (see *Stability and dissolution of Ba and Na in the test medium* section).

The treatment concentrations used in the Ba and NaCl chronic exposures were guided by acute toxicity data in the literature for *Daphni*a spp. (Tables 1 and 2). For single toxicant tests, dissolved Ba test concentrations were a 2.0 geometric series starting from 0.5 mg/L to 16 mg/L (online supplementary material Table S2 lists treatments). Sodium chloride was at 205 mg/L intervals greater relative to the modified MHW-Cl control water, up to 1,435 mg/L (online supplementary material Table S2 lists treatments, NaCl intervals were initially based on 500 µS/cm increments) because Na^+^ and Cl^-^ ions are present from the MHW-Cl salts (NaHCO_3_, CaCl_2_⋅2H_2_O and MgCl_2_⋅6H_2_O and KCl).

**Table 1.** Dissolved barium acute toxicity (≤ 48 hr) to *Daphnia* or c*eriodaphnia* spp

Phylum/subphylum	Scientific name	Organism life stage	Organism age	Barium salt used	Testing media/water	Exposure type	Effect	Test end-point	Ba^2+^ (mg/L)	Reference
**Crustacea**	*Ceriodaphnia dubia*	Neonate	< 24 hr	Cl_2_	SSW-Cl	Static	Mortality	48 hr EC10	14	(Golding et al., 2018)
		Neonate	< 24 hr	Cl_2_	SSW-Cl	Static	Mortality	48 hr EC20	15	(Golding et al., 2018)
		Neonate	< 24 hr	Cl_2_	SSW-Cl	Static	Mortality	48 hr EC50	17	(Golding et al., 2018)
		Neonate	< 24 hr	Cl_2_	DMW	Static	Mortality	48 hr EC10	28	(Golding et al., 2018)
		Neonate	< 24 hr	Cl_2_	DMW	Static	Mortality	48 hr EC20	31	(Golding et al., 2018)
		Neonate	< 24 hr	Cl_2_	DMW	Static	Mortality	48 hr EC50	38	(Golding et al., 2018)
	*Daphnia magna*	Neonate	< 24 hr	N.S.	Lake Superior Water	Static	Mortality	48 hr LC50	14.5	(Biesinger & Christensen, 1972)
		N.S.	N.S.	SO_4_	Tubewell hard water	Static	Mortality	24 hr EC50	52.82	(Khangarot & Ray, 1989)
		N.S.	N.S.	SO_4_	Tubewell hard water	Static	Mortality	48 hr EC50	32	(Khangarot & Ray, 1989)
		Neonate	< 24 hr	N.S.	Deionised reconstituted well water	Static	Mortality	24 hr LC50	>530	(LeBlanc, 1980)
		Neonate	< 24 hr	N.S.	Deionised reconstituted well water	Static	Mortality	48 hr LC50	410	(LeBlanc, 1980)
		Neonate	< 24 hr	(NO_3_)_2_	Standard reference water	Static	Immobilization	24 hr EC50	70.3	(Lilius et al., 1995)
		Neonate	< 24 hr	Cl_2_	Activated charcoal filtered tap water	Static	Immobilization	48 hr EC50	11	(Okamoto et al., 2015)

*Note:* N.S. = not specified; SSW-Cl = synthetic soft water with chloride instead of sulfate; DMW = diluted mineral water containing low sulfate

This table was populated using select results from the United States Environmental Protection Agency (USEPA) Ecotox Knowledgebase (USEPA, 2023) in addition to literature searches.

**Table 2.** Sodium chloride (NaCl) acute toxicity (≤ 48 hr) to *Daphnia* or C*eriodaphnia* spp

Phylum/subphylum	Scientific name	Organism life stage	Organism age	Exposure type	Effect	Test end-point	NaCl (mg/L)	Reference
**Crustacea**	*Ceriodaphnia dubia*	Neonate	< 24 hr	Static	Mortality	48 hr LC50	1,068	(Elphick et al., 2011)
		Neonate	N.S.	Static	Mortality	48 hr LC50	1,590	(Harmon et al., 2003)
		Neonate	< 24 hr	N.S.	Mortality	48 hr LC50	736	(Hoke et al., 1992)
		Neonate	< 24 hr	N.S.	Mortality	48 hr LC50	836	(Hoke et al., 1992)
		Neonate	< 24 hr	Static	Mortality	48 hr LC50	4,700	(Kszos et al., 2003)
		Neonate	< 24 hr	Static	Mortality	48 hr LC50	1,042	(Mount & Gulley, 1992)
		Neonate	< 24 hr	Static	Mortality	24 hr LC50	3,380	(Mount et al., 1997)
		Neonate	< 24 hr	Static	Mortality	48 hr LC50	1,960	(Mount et al., 1997)
		Neonate	< 24 hr	Static	Mortality	48 hr LC50	1,357	(Soucek et al., 2011)
		Neonate	< 24 hr	Static	Mortality	48 hr LC50	1,836	(Soucek et al., 2011)
		Neonate	< 24 hr	Static	Mortality	48 hr LC50	861	(Soucek et al., 2011)
		Neonate	< 24 hr	Static	Mortality	48 hr LC50	1,192	(Soucek et al., 2011)
		Neonate	< 24 hr	Static	Mortality	48 hr LC50	1,589	(Soucek et al., 2011)
		Neonate	< 24 hr	Static	Mortality	48 hr LC50	1,779	(Soucek et al., 2011)
		Neonate	< 24 hr	Static	Mortality	48 hr LC50	1,489	(Soucek et al., 2011)
		Neonate	< 24 hr	Static	Mortality	48 hr LC50	1,402	(Soucek et al., 2011)
		Neonate	< 24 hr	Static	Mortality	48 hr LC50	1,356	(Soucek et al., 2011)
		Neonate	< 24 hr	Static	Mortality	48 hr LC50	977	(Soucek et al., 2011)
		Neonate	< 24 hr	Static	Mortality	48 hr LC50	1,249	(Soucek et al., 2011)
		Neonate	< 24 hr	Static	Mortality	48 hr LC50	1,154	(Soucek et al., 2011)
		Neonate	< 24 hr	Static	Mortality	48 hr LC50	1,317	(Soucek et al., 2011)
		Neonate	< 24 hr	N.S.	Mortality	48 hr LC50	2,330	(Valenti et al., 2007)
	*Daphnia ambigua*	Neonate	N.S.	Static	Mortality	48 hr LC50	2,000	(Harmon et al., 2003)
	*Daphnia magna*	Neonate	< 24 hr	Renewal	Mortality	48 hr LC50	4,745	(Arambašić et al., 1995)
		Neonate	<12 hr	Static	Mortality	48 hr LC50	1,640	(Biesinger & Christensen, 1972)
		Neonate	<12 hr	Static	Mortality	48 hr LC50	1,820	(Biesinger & Christensen, 1972)
		Neonate	< 24 hr	Static	Mortality	48 hr LC50	3,137	(Davies & Hall, 2007)
		Neonate	< 24 hr	Static	Mortality	48 hr LC50	3,222	(Davies & Hall, 2007)
		Neonate	< 24 hr	Static	Mortality	48 hr LC50	3,136	(Davies & Hall, 2007)
		N.S.	N.S.	Static	Mortality	24 hr LC50	3,412	(Dowden & Bennett, 1965)
		N.S.	N.S.	Static	Mortality	∼24 hr LC50	6,447	(Dowden & Bennett, 1965)
		N.S.	N.S.	Static	Mortality	48 hr LC50	3,310	(Dowden & Bennett, 1965)
		Multiple	N.S.	Static	Mortality	24 hr LC50	3,412	(Dowden, 1961)
		Multiple	N.S.	Static	Mortality	48 hr LC50	3,318	(Dowden, 1961)
		Neonate	< 24 hr	Static	Mortality	48 hr LC50	3,630	(Elphick et al., 2011)
		Neonate	< 24 hr	N.S.	Mortality	48 hr LC50	5,009	(Hoke et al., 1992)
		Neonate	< 24 hr	Static	Mortality	48 hr LC50	1,560	(Kszos et al., 2003)
		Neonate	< 24 hr	Static	Mortality	48 hr LC50	6,027	(MacLean et al., 1989)
		Neonate	< 24 hr	Static	Mortality	48 hr LC50	6,027	(MacLean et al., 1989)
		Neonate	< 24 hr	Static	Mortality	48 hr LC50	5,600	(MacLean et al., 1989)
		Neonate	< 24 hr	Static	Mortality	48 hr LC50	5,600	(MacLean et al., 1989)
		Neonate	< 24 hr	Static	Mortality	48 hr LC50	5,020	(MacLean et al., 1989)
		Neonate	< 24 hr	Static	Mortality	48 hr LC50	5,480	(Martínez-Jerónimo & Martínez-Jerónimo, 2007)
		Neonate	< 24 hr	Renewal	Mortality	48 hr LC50	6,500	(Meyer et al., 1985)
		Neonate	< 24 hr	Static	Mortality	24 hr LC50	6,380	(Mount et al., 1997)
		Neonate	< 24 hr	Static	Mortality	48 hr LC50	4,770	(Mount et al., 1997)
		Neonate	< 24 hr	N.S.	Mortality	48 hr LC50	4,960	(Valenti et al., 2007)
	*Daphnia pulex*	Neonate	24–48 hr	Static	Mortality	24 hr LC50	4,170	(Bezirci et al., 2012)
		Neonate	24–48 hr	Static	Mortality	24 hr LC50	5,090	(Bezirci et al., 2012)
		Neonate	24–48 hr	Static	Mortality	48 hr LC50	3,320	(Bezirci et al., 2012)
		Neonate	24–48 hr	Static	Mortality	48 hr LC50	4,050	(Bezirci et al., 2012)
		N.S.	N.S.	Static	Mortality	48 hr LC50	3,050	(Birge et al., 1985)
		N.S.	N.S.	Static	Mortality	48 hr LC50	1,470	(Birge et al., 1985)
		Neonate	N.S.	N.S.	Mortality	48 hr LC50	1,812	(Gardner & Royer, 2010)
		Neonate	N.S.	N.S.	Mortality	48 hr LC50	2,042	(Gardner & Royer, 2010)
		Neonate	N.S.	Renewal	Mortality	48 hr LC50	1,910	(Robison, 2011)
		Neonate	N.S.	Renewal	Mortality	48 hr LC50	1,870	(Robison, 2011)
		Neonate	N.S.	Renewal	Mortality	48 hr LC50	2,210	(Robison, 2011)
		Neonate	N.S.	Renewal	Mortality	48 hr LC50	1,760	(Robison, 2011)
		Neonate	N.S.	Renewal	Mortality	48 hr LC50	2,480	(Robison, 2011)

*Note:* N.S. = not specified

This table was populated using select results from the United States Environmental Protection Agency (USEPA) Ecotox Knowledgebase (USEPA, 2023).

In the binary Ba and NaCl test, dissolved Ba concentrations were tested at 1.5, 3, 6, and 12 mg/L (4 concentrations) combined with 410, 810, and 1,230 mg/L (three concentrations) additional NaCl relative to the modified MHW-Cl control water (online supplementary material Table S2 lists treatments, NaCl intervals were initially based on 1,000 µS/cm relative to the control). This resulted in twelve binary Ba and NaCl treatments and the overall control treatment consisting of the modified MHW-Cl. The Ba concentrations were selected based on an initial approximate reproductive EC50 of 12 mg/L from chronic single-chemical testing. The Ba, Na^+^, and Cl^-^ concentrations are commonly found within untreated or undiluted coal bed methane (CBM) wastewater (Willems et al., 2022) and diluted SG wastewater if spilled or leaked into surface waters, where significant dilutions (10 to 100s of fold are expected).

### Stability and dissolution of Ba and Na in the test medium

Water samples (50 m) were collected from freshly prepared excess treatment solution (new, 0 hr) or pooled from three to four replicates (old, 24 hr) from each active treatment at the start and end of each chronic test. Each sample was firstly 0.45 µm syringe filtered and then 0.1% v/v acidified with concentrated nitric acid (HNO_3_ 70%). A select set of samples from these three tests was then analyzed by the National Measurements Institute (NMI) Australia (a National Association of Testing Authorities, Australia [NATA] accredited laboratory) via their in-house method NT2.47. This utilized inductively coupled plasma atomic emission spectrometry and inductively coupled plasma mass spectrometry to evaluate dissolved Ba and sodium concentrations (see *Water chemistry* section).

### Data analysis

A descriptive statistical approach using IBM SPSS Statistics Ver. 24 for Mac OS (IBM Corp., 2016) was used to perform a one-way analysis of variance (ANOVA) with Tukey’s post hoc test for multiple groups of comparison to assess significant (α  ≤  .05) differences in reproduction between groups, within each of the three chronic tests, where *n* = 10 for each treatment group.

In NaCl treatments, measured electrical conductivity (µS/cm) was corrected to mg/L using analytically determined (inductively coupled plasma atomic emission spectrometry) concentrations of Na^+^ to determine the total added NaCl (mg/L) content. This resulted in a 0.41x factor (mean ± SE of 0.41 ± 0.006) for converting µS/cm into mg/L, comparable to Willems et al. (2023). This was to ensure standardized units for both toxicants and to enable comparisons to other studies that report NaCl as mg/L.

**Figure 1.**
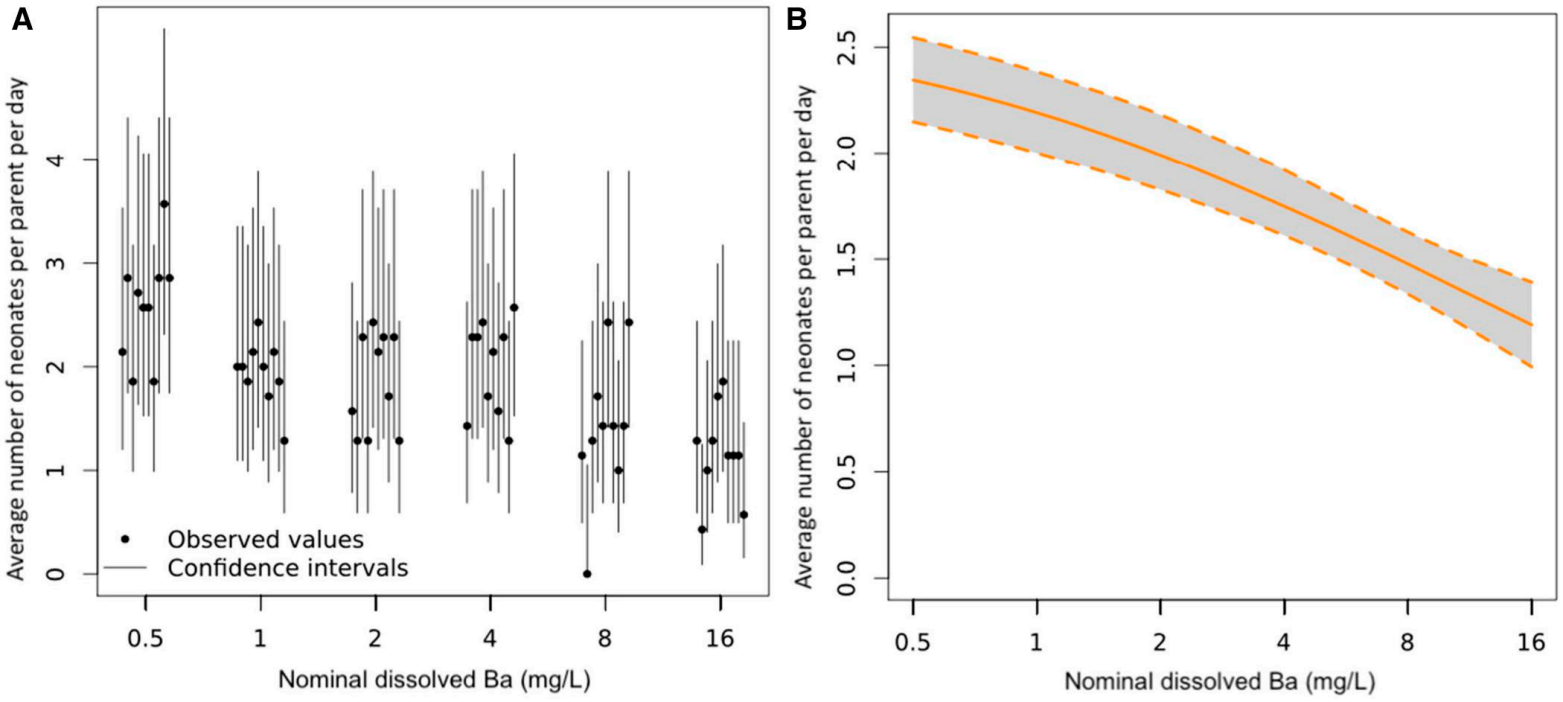
*Ceriodaphnia dubia* reproduction at the end of the 7-day dissolved barium (Ba) exposure. (A) Fraction of surviving individuals at the end of the bioassay as a function of the concentration of the contaminant (dissolved Ba). Vertical lines represent 95% binomial confidence intervals for the estimated fraction. (B) Mean number of offspring per individual-day as a function of concentration, with the associated 95% credible interval in light grey. See Table S6 for complete reproduction data by replicate and time (day) series. These figures were produced using the online tool *MOdeling and StAtistical tools for ecotoxICology* (MOSAIC; Charles et al., 2017).

**Figure 2.**
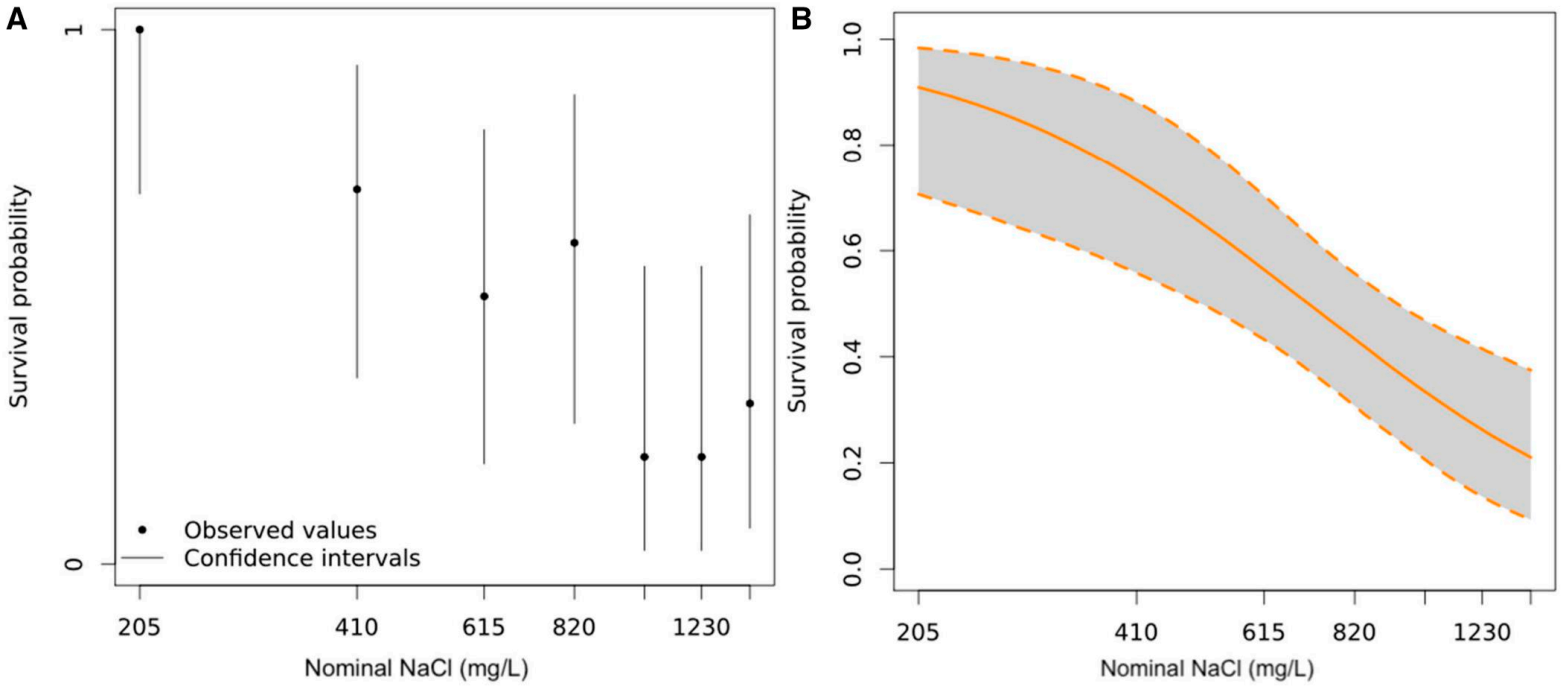
*Ceriodaphnia dubia* parent survival at the end of the 6-day sodium chloride (NaCl) exposure. (A) Fraction of surviving individuals at the end of the bioassay as a function of the concentration of the contaminant (NaCl). Vertical lines represent 95% binomial confidence intervals for the estimated fraction. (B) Fitted survival probability log plot with 95% credible intervals in grey. See Table S7 for complete parent survival data by replicate and time (day) series. These figures were produced using the online tool *MOdeling and StAtistical tools for ecotoxICology* (MOSAIC; Charles et al., 2017). NaCl is at concentrations additional to the control as Na+ and Cl^-^ ions are present in moderately hard water chloride (MHW-Cl) salts.

**Figure 3.**
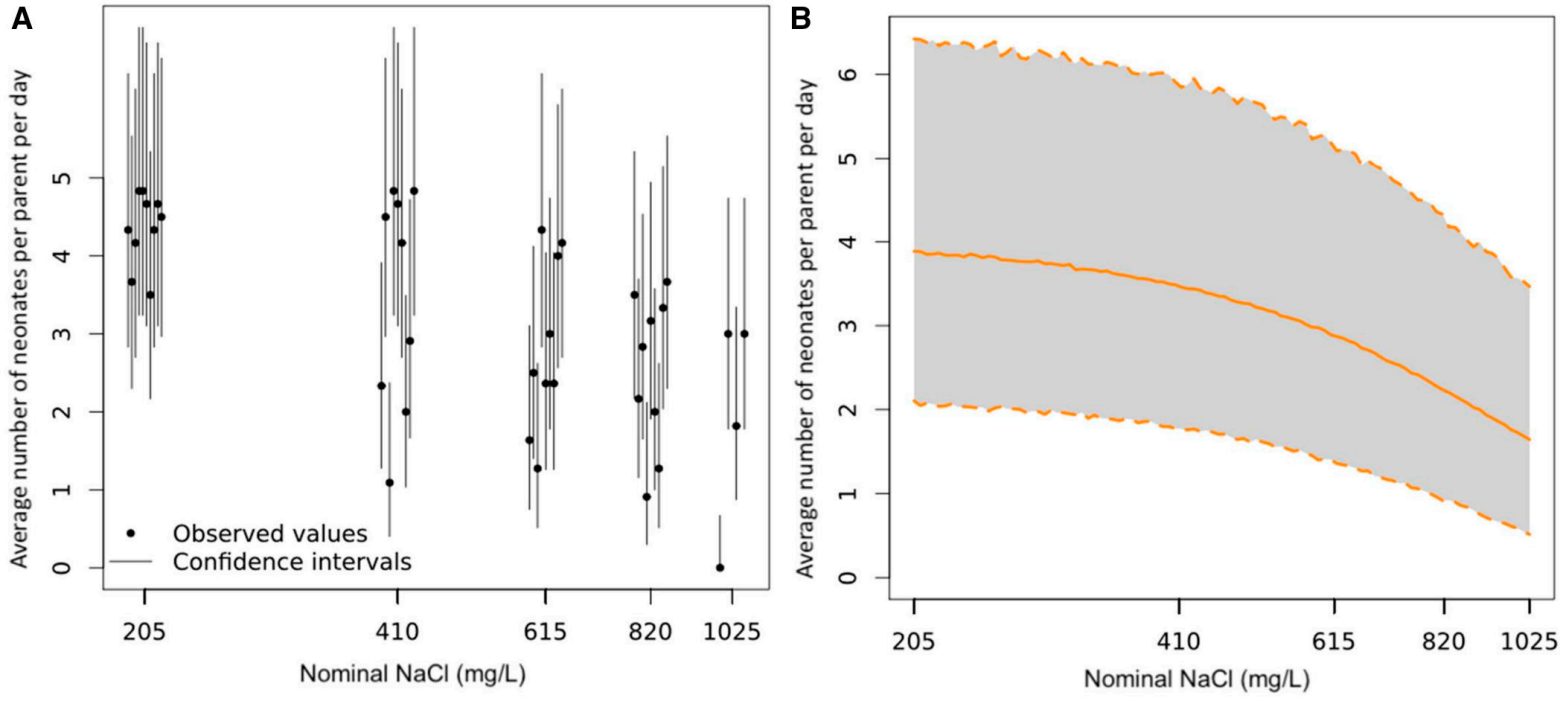
*Ceriodaphnia dubia* reproduction at the end of the 6-day sodium chloride (NaCl) exposure. (A) Fraction of surviving individuals at the end of the bioassay as a function of the concentration of the contaminant (NaCl). Vertical lines represent 95% binomial confidence intervals for the estimated fraction. (B) Mean number of offspring per individual-day as a function of concentration, with the associated 95% credible interval in light grey. See Table S7 for complete reproduction data by replicate and time (day) series. These figures were produced using the online tool *MOdeling and StAtistical tools for ecotoxICology* (MOSAIC; Charles et al., 2017). (1) NaCl is at concentrations additional to the control as Na^+^ and Cl^-^ ions are present in moderately hard water chloride (MHW-Cl) salts. (2) This figure has used reproductive data that is not confounded by high mortality. It excluded the 1,230 and 1,435 mg/L NaCl treatments and omitted any replicates across other treatments (control and 205 to 1,025 mg/L NaCl treatments) where *C. dubia* parents died on day 5 or earlier.

**Figure 4.**
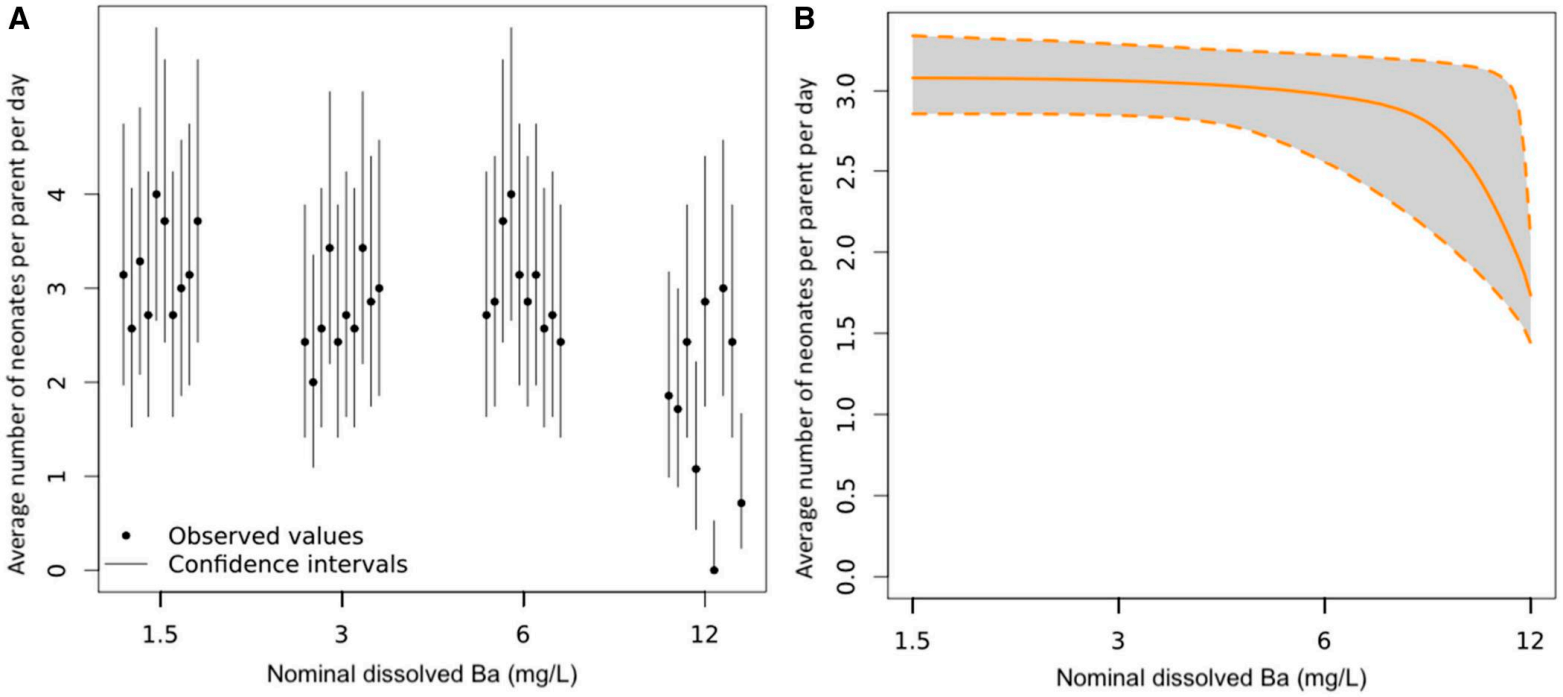
*Ceriodaphnia dubia* reproduction at the end of the 7-day binary exposure to 7-day 410 mg/L sodium chloride (NaCl) combined at 1.5, 3, 6, and 12 mg dissolved Ba/L. (A) Fraction of surviving individuals at the end of the bioassay as a function of the concentration of the contaminants (dissolved barium [Ba] and NaCl). Vertical lines represent 95% binomial confidence intervals for the estimated fraction. (B) Mean number of offspring per individual-day as a function of concentration, with the associated 95% credible interval in light grey. See Table S8 for complete reproduction data by replicate and time (day) series for all binary combinations of dissolved Ba and NaCl. These figures were produced using the online tool *MOdeling and StAtistical tools for ecotoxICology* (MOSAIC; Charles et al., 2017). (1) The 410 mg/L NaCl concentration is in addition to the control, as Na^+^ and Cl^-^ ions are present in moderately hard water chloride (MHW-Cl) salts. (2) Figure 4 uses reproductive data that is not confounded by high parent mortality. This was done by excluding replicates from analysis where *C. dubia* parents had died on day 6 or earlier.

An online statistical tool *MOdeling and StAtistical tools for ecotoxICology* (MOSAIC; Charles et al., 2017), which utilizes a Bayesian approach, was used for data analysis. The MOSAIC “Surv(ival) Standard” tool was used to determine EC endpoints and associated 95% credible interval (CI) estimates (where applicable) for parent survival lethal effect (LC) concentrations. The reproduction tool from MOSAIC was used to calculate estimated reproduction EC values and associated 95% CIs (where applicable). Figures 1–4 are log-transformed along the x-axis; hence, no control (0 mg/L) treatment appears. Data associated with the binary testing (dissolved Ba and NaCl) were analyzed through these two tools by splitting into three data sets based on NaCl concentrations at 410, 820, and 1,230 mg/L. It should be noted that reproduction data using MOSAIC is calculated to determine the number of offspring per animal-day (i.e., the average number of neonates produced for each day the parent is alive) and takes into consideration parent mortality, where survival data in the dataset is used to calculate the effective period of observation for which each animal may have reproduced (Charles et al., 2017). Further data refinement was performed as detailed below to reduce the confounding effects of parent mortality on reproduction in both the NaCl-only and binary dissolved Ba and NaCl tests.

For the NaCl reproduction data set (online supplementary material Table S7), confounding effects of parent mortality on reproduction were refined to remove the 1,230 and 1,435 mg/L NaCl treatments, and the remaining treatments had any replicates omitted where parents had died by Day 5 (out of 6 days of exposure) or earlier.

For the binary exposure of dissolved Ba and NaCl to *C. dubia* reproduction (online supplementary material Table S8), the confounding effects of early parent mortality on reproduction were eliminated by removing replicates where parents had died by Day 6 (out of 7 days for this test duration) to determine the Ba EC10, EC20, and EC50 at 410 mg/L NaCl. No treatments required outright exclusion, as parent survival was not as severely reduced as the higher treatments in the NaCl-only toxicity test (see *Impacts of NaCl exposure on reproduction* section).

## Results and discussion

### Water chemistry

Dissolved Ba concentrations ranged from 90%–110% of nominal (online supplementary material Table S1), except for the 0.5 mg dissolved Ba/L, which was 80% of the nominal. Dissolved Ba was also measured in the binary Ba and NaCl test at 1.5 and 12 mg dissolved Ba/L in combinations with 410 and 1230 mg/L NaCl, and recoveries based on nominal concentrations were 105%–110% (not shown). These dissolved Ba recoveries are similar to those of Golding et al. (2018) with their synthetic soft water chloride (SSW-Cl) via modeling (visual MINTEQ), where they estimated that more than 98% of the total Ba would be dissolved Ba at concentration ranges comparable with this study. We reiterate that substituting of ${\text{SO}}_{4}^{2-}$ containing salts is essential when assessing dissolved Ba toxicity, as these two ions react, forming the highly water-insoluble BaSO_4_. Golding et al. (2018) also showed that without substituting ${\text{SO}}_{4}^{2-}$ in SSW, added total Ba from 0.1 to 100 mg/L was only predicted to increase as dissolved Ba from 0.032 to 0.19 mg/L.

Water quality parameters were measured at the beginning (0 hr new and 24 hr old) and end (24 hr before test termination (0 hr new) and at test termination (24 hr old) in each of the three chronic tests (online supplementary material Table S2) in newly prepared (0 hr) and old (24 hr) solutions in each treatment. Across testing, the incubator temperature was 25 ± 1°C, DO was 8.2 ± 0.6 mg/L, pH was 8.0 ± 0.7, and electrical conductivity of the modified MHW-Cl was 540 ± 40 µS/cm; within an experiment, the MHW-Cl conductivity variability was up to ± 25 µS/cm. Where NaCl was used, variability from nominal conductivity was usually < 3%. The modified MHW-Cl bulk stock solution drifted more than 10% throughout the test due to evaporation from constant aeration despite the carboy containing the modified MHW-Cl solution being covered.

### Chronic toxicity of Ba to water fleas

#### Impacts of dissolved Ba exposure on parent survival

There was no mortality concentration-effect response from the parent *C. dubia* exposed to 0.5 to 16 mg dissolved Ba/L (online supplementary material Table S6), with only a single parent experiencing mortality at 8 mg dissolved Ba/L across all treatments. Therefore, it was not possible to determine the LC responses of parent *C. dubia* for dissolved Ba. The 0.5 to 16 mg/L dissolved Ba concentrations in this study were comparable with those within untreated CBM FPW with an approximate average of 25 mg dissolved Ba/L, although also commonly found at lower concentrations (5–20 mg dissolved Ba/L). The concentrations tested are still much lower than those in untreated/raw SG FPW (approximate average of 815 mg dissolved Ba/L; Willems et al., 2022). If these SG FPW accidentally enter surface water via a spill or leak from transporting or holding of these waters (Patterson et al., 2017), they could be at concentrations relevant to those tested in this study.

#### Impacts of dissolved Ba exposure on reproduction

Relative to the control, reproduction (the number of total neonates at the end of Day 7 per original parent, online supplementary material Figure S1A) was significantly reduced at 2 mg dissolved Ba/L (*p* = .015) and highly significantly reduced (*p* ≤ .001) at 8 and 16 mg dissolved Ba/L (online supplementary material Table S3A, Figure 1), with an estimated dissolved Ba reproductive EC20 and EC50 of 0.95 and 10.1 mg Ba/L respectively (Table 3). The calculated reproductive EC10 estimate was omitted and did not align with our reproductive data. This is attributed to similar reproduction between the control group and 0.5 mg dissolved Ba/L. Then, there was a sharp drop in reproduction to 1 mg dissolved Ba/L, with the 2 and 4 mg dissolved Ba/L also having reproductive outputs similar to each other and the 1 mg dissolved Ba/L treatment, skewing the data.

**Table 3.** Summary of mortality (parents) and reproduction (number of neonates) effect concentration (EC; mg/L) values and (95% credible intervals) of dissolved Ba and NaCl single chemical exposures for *Ceriodaphnia dubia*.

Toxicant and respective b and d values	Duration (days)	Lethal effect concentration			Reproduction effect concentration		
		LC10	LC20	LC50	EC10	EC20	EC50
**Dissolved Ba**	7	N.D.	N.D.	N.D.	N.R.	0.95 [0.19 - 3.22]	10.1 [5.61 - 17.8]
**Effect intensity (b)**		N.D.			0.59 [0.367 - 0.976]		
**Control value (d)**		N.D.			2.76 [2.40 - 3.12]		
**NaCl**	6	N.R.	335 [116–507]	708 [502–939]	365 [149–651]	510 [282–760]	903 [738–1120]
**Effect intensity (b)**		1.88 [0.837–3.12]			2.43 [1.24–5.78]		
**Control value (d)**		N.A.			4.13 [3.59–4.70]		

*Note:* Effect intensity (b) and control value (d) log-logistic model parameters are also reported (denoted these letters in MOSAIC) for the respective LC or EC endpoints where applicable and their [95% credible intervals].

N.D. = Not determinable. As only 1 adult *C. dubia* died (at 8 mg/L) across the whole barium chronic exposure at 2.0 factor geometric series from 0.5 to 16 mg dissolved Ba/L.

N.R = Not representative. Based on the raw data the estimated LC/EC endpoint values from MOSAIC did not closely represent the observed responses in our data.

N.A. = Not applicable. Due to 100% survival in the control treatment for the NaCl only exposure.

There is a paucity of Ba ecotoxicity data, even in acute toxicity water flea data (Table 1), and even less in chronic toxicity data across freshwater biota, including water fleas (online supplementary material Table S4), so sparse chronic comparisons can be made. Our reproductive EC50 of 10.1 mg dissolved Ba/L is similar to the LC50 of 13.5 mg dissolved Ba/L found by Biesinger & Christensen 1972; online supplementary material Table S4), which utilized a 21-day chronic exposure with *D. magna* using a sample of Lake Superior water as dilution water. It is unknown whether there were some potential confounding effects of sulfate being present as they used a natural water source, and sulfate was not measured in their study.

### Ba toxicity

Dissolved Ba toxicity to water fleas is highly dependent on its solubility. It is influenced by the presence of other anions, such as sulfate (${\text{SO}}_{4}^{2-}$) and, to a lesser extent, carbonate (${\text{CO}}_{3}^{2-}$) ions (Golding et al., 2018). Thus, the choice of Ba salt (e.g., BaCl_2_, BaSO_4,_ or BaCO_3_) is important when performing a toxicity assessment with Ba. Sulfate and carbonate are also present in some salts that constitute standard MHW (ASTM International, 2014). Thus, a particular effort needs to be made to substitute the ${\text{SO}}_{4}^{2-}$ containing salts with Cl containing varieties, as BaSO_4_ is highly water-insoluble (Golding et al., 2018). The use of natural water sources as dilution waters for exposure treatments may also contain ${\text{SO}}_{4}^{2-}$, and the use of these natural waters in combination with Ba toxicity assessment should be measured before being used. Synthetic waters like MHW-Cl can eliminate the potential confounding impacts of sulfate (Verbruggen et al., 2020).

Dissolved Ba is a physiological antagonist for K^+^, blocking passive efflux of the potassium channels, leading to cells becoming K^+^ deficient, as dissolved Ba blocks K^+^ channels of the (Na^+^, K^+^)‐ATPase pump in cell membranes. This results in reduced excitability of muscle fibers, which can cause muscle paralysis and death (Bhoelan et al., 2014; Oskarsson, 2015). In the environment, animals affected by paralysis will become increasingly lethargic and easier prey. The (Na^+^, K^+^)‐ATPase pumps are also essential in facilitating the secondary transport of various molecules (amino acids, metabolites, neurotransmitters, and sugars) and other ions (H^+^, Ca^2+^, Cl^−^; Clausen et al., 2017). Thus, dissolved Ba-induced changes in these pumps could reduce essential nutrients for the reproduction of the parent *C. dubia*. The Ca^2+^ ion is especially important for growth associated with carapace molting (Pérez-Fuentetaja & Goodberry, 2016; Tan & Wang, 2010). Pérez-Fuentetaja and Goodberry (2016) showed that reduced Ca^2+^ adversely affected reproduction, molting, and population growth in *Daphnia pulex*.

There are currently no freshwater Ba Australian default guideline values (Australian and New Zealand Guidelines [ANZG], 2018) due to a combination of sparse dissolved Ba-associated ecotoxicity literature and the significant causes of inconsistencies between studies, as reflected in Table 1. In The Netherlands, there are long-term or annual average environmental quality standards (AA-EQS) of 0.093 mg dissolved Ba/L (reported as 93 µg dissolved Ba/L), while short-term or maximum acceptable concentration environmental quality standards at 1.1 mg dissolved Ba/L (Verbruggen et al., 2020). The annual average environmental quality standards value of 0.093 mg dissolved Ba/L is approximately tenfold lower than this study's reported reproductive EC20 of 0.95 mg dissolved Ba/L (Table 3). In the environment, there could be varying levels of sulfate present in surface waters. It would be important to not just develop dissolved Ba guidelines but also precipitated Ba guidelines, beginning with BaSO_4_ due to its very low solubility in water. Organisms at different trophic levels have shown varying sensitivity to dissolved and precipitated forms of Ba due to underlying modes of toxicity, as discussed in Golding et al. 2018 and summarized later in this section. Surface waters with low sulfate concentrations affected by inorganically rich SG FPW, which are likely to have highly elevated dissolved Ba (Willems et al., 2022), will have both precipitated and dissolved Ba, and thus, organisms in these surface waters can be affected by both dissolved and precipitated forms of Ba. Dissolved Ba guideline values would be suitable for freshwaters with low ${\text{SO}}_{4}^{2-}$. However, further research would be needed to provide threshold ${\text{SO}}_{4}^{2-}$concentration values to guide researchers on when to use a future-developed dissolved Ba or precipitated Ba as BaSO_4_ guideline values.

Multiple studies have measured Ba in proximal surface waters affected by discharges or spills from unconventional oil and gas (Bonetti et al., 2021; Lauer et al., 2016; McLaughlin et al., 2020; Ni et al., 2022; Warner et al., 2013). These studies consistently showed that Ba concentrations were diluted downstream (where applicable) from the source of spill or discharge of the FPW. Warner et al. (2013) investigated an effluent discharge point from a brine treatment plant and measured mean dissolved Ba at 13.4 mg/L (*n* = 18), whereas 20–300 m downstream average concentrations had declined to 0.93 mg/L (*n* = 7). This average downstream concentration of 0.93 mg/L is similar to our reproductive EC20 of 0.95 mg dissolved Ba/L (Table 3). Surface water regions closer to discharge points or leaks/spills from untreated FPW are at greater risk. Highly saline and inorganic-rich SG FPW will have increased Ba toxicity risk compared with those from CBM FPW (Willems et al., 2022).

Greater Ba concentrations required to elicit a particular response (e.g., LC/EC50) are indicative of Ba being mostly present in a less bioavailable precipitated form (Golding et al., 2018) such as BaSO_4_ to *Daphnia* spp (Table 1). However, the green microalga *Chlorella* sp. 12 was considerably more sensitive to BaSO_4_ precipitate than dissolved Ba (Golding et al., 2018). This affects primary productivity in freshwater ecosystems with increased ${\text{SO}}_{4}^{2-}$ and if a spill or leak of Ba-rich SG FPW enters these systems. It could result in isolated regions with reduced primary productivity, indirectly affecting primary consumers like water fleas and organisms above them in food webs.

### Chronic toxicity of NaCl to water fleas

#### Impacts of NaCl exposure on parent survival

No *C. dubia* parents had died in either the control or 205 mg/L NaCl, whereas mortality increased at 410 mg/L and above (online supplementary material Table S6, Figure 2). The NaCl LC20 and LC50 for *C. dubia* were estimated to be 335 mg/L and 708 mg/L, respectively (Table 3). An LC10 was estimated using MOSAIC, but its value 217 (45.5–384) was nearly 205 mg/L, the treatment that had 100% survival.

The arithmetic series of NaCl concentrations from 205 mg/L intervals up to 1,435 mg/L relative to the control (or 500 µS/cm NaCl intervals up to 3500 µS/cm NaCl) were at concentrations found in CBM FPW or that are typically greatly exceeded within SG FPW due to hypersalinity or even brine-like properties (Willems et al., 2022).

#### Impacts of NaCl exposure on reproduction

Reproduction (the number of total neonates at the end of Day 6 per original parent) had significantly declined from ≥ 820 mg/L NaCl (*p* < 0.05, online supplementary material Table S3B). In the two highest NaCl treatments (1,230 and 1,435 mg/L), the early and high levels of parent mortality adversely affected reproduction (online supplementary material Figure S1B, Table S7). Using refined data to account for the high rate of parent mortality in this NaCl-only exposure (see *Data analysis* section), the estimated NaCl reproductive endpoints of EC10, EC20, and EC50 were 365 mg/L, 510 mg/L, and 903 mg/L, respectively (Table 3, Figure 3).

Sodium chloride is commonly used as a reference toxicant, with widespread literature reporting both acute (Table 2) and chronic responses (online supplementary material Table S5) in water fleas, amongst other organisms. Harmon et al. (2003), also using *C. dubia*, had reported a 7-day reproductive EC50 of 1350 mg/L NaCl. A factor of approximately 1.5-fold greater than the 6-day reproductive EC50 of 903 mg/L NaCl (Table 3). These differences could be attributed to the water used. In this study, our water was modified to prevent the presence of sulfate, whereas Harmon et al. (2003) used standard MHW, as in their study; there was no need to remove sulfate for Ba solubility issues. Another major factor could be the food source, where Harmon et al. (2003) had used a modified yeast, Cerophyl, and trout chow combination feed in addition to including *Raphidocelis subcapitata* (reported as a former name *Selenastrum capricornutum*) and alfalfa, compared with this study (see *Test organism and culture conditions* section).

The control had a mean of 22.8 neonates per parent across the test (online supplementary material Table S7) or 3.8 neonates per day per parent across the 6-day test. In comparison, the 205 mg/L NaCl treatment had a mean of 26.1 neonates or 4.35 neonates per day per parent across the 6-day test (the control and 205 mg/L NaCl treatments had no parent mortality). However, this was determined to be a nonsignificantly different (*p* > .05, online supplementary material Table S3B) increase in reproduction in the 205 mg/L NaCl treatment compared with the control. In the environment, slightly elevated NaCl may increase populations, shifting species composition and abundance. This response of increased (nonsignificant) reproduction could be beneficial to *C. dubia*, or this relatively slight increase might have caused a hormesis response and increased reproductive output before further additions of NaCl (≥ 410 mg/L; online supplementary material Figure S1B, Figure 3) caused greater osmoregulatory stress, directly reducing reproduction and increasing parent mortality.

### NaCl toxicity

A comparison can be made between the NaCl reproductive endpoints (Table 3) and the mg Cl^-^/L chronic guideline values of 230 mg Cl^-^/L (USEPA, 1988) and long-term values for protection (chronic) of aquatic life at 120 mg Cl^-^/L (Canadian Council of Ministers of the Environment, 2011). The reproductive NaCl EC10 of 365 mg/L (Table 3) would be equivalent to 221 mg Cl^-^/L (assuming Cl is 60.6% mass of NaCl). The set guideline values of 230 mg Cl^-^/L and 120 mg Cl^-^/L would provide reasonably high protection for reproductive impacts for the salinity (as NaCl) sensitive *C. dubia.* These water fleas are usually more sensitive to changes in water quality than other large water fleas (*Daphnia magna*), because *C. dubia* are small in size (≤ 1 mm length) and thus have greater surface area to volume ratios and experience more profound osmotic stresses, indicated by the lower NaCl concentrations that elicit a 48 hr LC50 response (Table 2).

Another key factor to consider is the major ion chemistry ratios (Mount et al., 2016), with water hardness influencing the toxicity of chloride (Soucek et al., 2011) and, thus, NaCl exposures. Increased water hardness as Ca and Mg increased the tolerance to chloride in *C. dubia* in acute tests (Soucek et al., 2011).

Studies that had investigated Ba in proximal surface waters affected by discharges or spills from unconventional oil and gas also typically investigated concentrations of Na^+^ and Cl^-^ (Lauer et al., 2016; McLaughlin et al., 2020; Ni et al., 2022; Warner et al., 2013). Lauer et al. (2016) found Na^+^ and Cl^-^ mean concentrations in FPW of 38,700 and 88,500 mg/L, respectively. The same study also found in proximal spill sites (i.e., situated near the origin of the spill point) separated by two classes of spills (type A and B, where type A are Bakken brines and type B are recycled Bakken brines). The average Na^+^ and Cl^-^ concentrations for spill type A were 3720 mg Na^+^/L and 7040 mg Cl^-^/L; spill type B were 1840 mg Na^+^/L and 5040 mg Cl^-^/L (Lauer et al., 2016). This results in approximate Na^+^ dilutions of tenfold to twentyfold and Cl^-^ dilutions of twelvefold to eighteenfold from the FPW into the surface waters. These diluted Na^+^ and Cl^-^ concentrations still greatly exceed our NaCl EC50 of 903 mg/L (Table 3).

### Binary Ba and NaCl chronic toxicity to water fleas

#### Impacts of binary dissolved Ba and NaCl exposure on parent survival

Across the 13 treatments (see *Chronic toxicity tests* section) in this binary test, parent survival remained above 50% (online supplementary material Table S8), with the lowest rate of parent survival observed in the 1.5 mg dissolved Ba/L combined with 1230 mg/L NaCl. Reliable survival lethal concentration data and associated plots could not be calculated due to similar levels of survival within the 410, 820, and 1,230 mg/L NaCl data sets, each having dissolved Ba tested at 1.5, 3, 6, and 12 mg/L (online supplementary material Table S8).

A significant shortcoming of this binary test was the absence of single chemical controls with no addition of dissolved Ba only or NaCl only (relative to the control) and using only an overall control. Another shortcoming was using only three NaCl concentrations (410, 820, and 1,230 mg/L) and four dissolved Ba concentrations (1.5, 3, 6, and 12 mg/L). A minimum of six concentrations (including a control) for each of the two chemicals would have enabled more meaningful and powerful statistical analysis, resulting in a more comprehensive 36-treatment setup, with the additional treatments favoring lower concentrations for greater confidence in LC/EC10 and LC/EC20 estimates. The concentration series for each Ba and NaCl could have been a geometric halving, where the highest concentrations tested for each of the two chemicals were based on the EC50 for dissolved Ba-only exposure (10.1 mg/L, Table 3) and NaCl-only exposure (903 mg/L, Table 3). We recognize in hindsight that such a design would have better enabled the determination of antagonistic, additive, or synergistic effects between these chemicals at environmentally relevant concentrations similar to those found within CBM and SGW FPW (Willems et al., 2022) and in proximal spill or discharge regions (see *Ba toxicity* and *NaCl toxicity* sections).

### Impacts of binary dissolved Ba and NaCl exposure on reproduction

Across treatments (online supplementary material Figure S1C, where *n* = 10 replicates), reproduction decreased with increasing NaCl concentrations (410, 820, and 1,230 mg/L). Compared with the control, only the six (*p* < .04) and 12 (*p* < .023) mg dissolved Ba/L each at 1,230 mg/L NaCl was significantly different (online supplementary material Table S3).

The only meaningful data from this binary test, given the shortcomings described in the *Impacts of binary dissolved Ba and NaCl exposure on parent survival* section, was the reproductive EC10, EC20, and EC50 for dissolved Ba in combination at 410 mg/L NaCl, which was 8.87, 10.1, and 12.3 mg dissolved Ba/L, respectively (Table 4, Figure 4). Reproduction EC estimates at 820 and 1,230 mg/L NaCl were deemed unreliable due to similar rates of reproduction in these treatment series (i.e., 820 or 1,230 mg/L NaCl each at 1.5, 3, 6 and 12 mg dissolved Ba/L, online supplementary material Table S3C). The Ba EC20 at 410 mg/L NaCl of 10.1 mg dissolved Ba/L (Table 4) is 10.6-fold greater than the Ba-only EC20 of 0.95 mg dissolved Ba/L (Table 3). The Ba EC50 at 410 mg/L NaCl of 12.3 mg dissolved Ba/L (Table 4) is 1.2-fold greater than the Ba-only EC50 of 10.1 mg dissolved Ba/L (Table 3). These results suggest that slightly increased NaCl at 410 mg/L inhibits Ba toxicity, particularly at lower concentrations of Ba with a much higher binary EC20 value than the Ba-only EC20. Mechanistically, this could be due to greater concentrations of Na^+^ or overall ionic composition (also including Cl^-^) that could reduce the ability of dissolved Ba to interact and disrupt normal functions of the (Na^+^, K^+^)‐ATPase pump in cell membranes (see *Ba toxicity* section).

**Table 4.** Summary of reproduction endpoints (number of neonates) effect concentrations (EC) for dissolved Ba (mg/L) and [95% credible intervals] for salinity as NaCl at 410 mg/L from the 7-day binary dissolved Ba and NaCl exposure using *Ceriodaphnia dubia*.

	Reproduction effect concentration (mg dissolved Ba/L)		
NaCl (mg/L)	EC10	EC20	EC50
**410**	8.87 [3.58–11.7]	10.1 [5.64–11.8]	12.3 [11.4–14.1]
**Effect intensity (b)**		6.31 [1.75–66.5]	
**Control value (d)**		3.08 [2.86–3.38]	

No survival lethal concentration endpoints were able to be reliably determined from the binary test data set due to either none or low similar parent mortality across limited treatments. Also reproduction (number of neonates) in the 820 and 1,230 mg/L NaCl data set either showed not to be significantly affected by additions barium (Table S3C). Effect intensity (b) and control value (d) log-logistic model parameters are also reported (denoted these letters in MOSAIC) for the respective EC endpoints where applicable and their [95% credible intervals].

### Binary toxicity interactions

Organisms in the environment are exposed to many chemicals in combination. Binary chemical toxicity assessment is used to begin understanding these chemical interactions. Metals (e.g., Ba) and salinity (as NaCl) combinations have generally been well studied and reviewed by Hall & Anderson (1995). They state that toxicity was reported to increase with decreased salinity for most metals, such as Cd, Cr, Cu, Hg, Ni, and Zn. This is likely related to the greater bioavailability of free metal ion forms, which are typically more toxic. However, there is still a paucity of research investigating binary toxicity of dissolved Ba and salinity, whether it be NaCl (Willems et al., 2023) or other forms, e.g., synthetic marine salts using freshwater invertebrates like *C. dubia*.

Other chemical and mechanistic interactions may exist that reduce the toxicity of Ba at relatively low NaCl (410 mg/L) concentrations. *C. dubia* has a calcium requirement, particularly during molting stages, and interference by dissolved Ba through the blocking of calcium membrane transport channels is another potential mechanism for toxicity (Golding et al., 2018). The increased Na^+^ and Cl^-^ concentrations in solution may reduce/prevent dissolved Ba from interacting with these calcium membrane transport channels. Thus, the toxicity of Ba was reduced at lower Ba concentrations like the EC20 (see *Impacts of binary dissolved Ba and NaCl exposure on reproduction* section). A normal functioning of these channels would allow more calcium, an essential nutrient in water flea reproduction (see *Ba toxicity* section), to be transported.

## Conclusion

This study presents the first data on the chronic toxicity of dissolved Ba to the water flea *C. dubia* without confounding effects or interactions from precipitated BaSO_4_. This medium was achieved by substituting ${\text{SO}}_{4}^{2-}$ containing salts from standard test mediums with those containing Cl^-^ (modified MHW-Cl) and using a highly water-soluble Ba salt (e.g., BaCl_2_). This study is also the first to evaluate chronic exposure effects of binary interactions of dissolved Ba with changing salinity (as NaCl), using reproduction as an endpoint. However, there were considerable shortcomings with the experimental design that should be revisited. Barium solubility is a crucial factor that must be considered when designing/planning toxicity tests with this element.

Flowback-produced waters from unconventional gas extraction have a high percentage of total Ba as dissolved Ba due to relatively low ${\text{SO}}_{4}^{2-}$ concentrations. This has important environmental implications when surface waters also have low ${\text{SO}}_{4}^{2-}$ concentrations, and untreated FPW is spilled or leaked into these surface waters. This enables Ba from the FPW to remain mostly as dissolved Ba, increasing bioavailability and thus toxicity to *C. dubia* and other similar organisms in freshwater ecosystems. However, it has been shown in other studies that the green microalga *Chlorella* sp. 12 is more sensitive to precipitated Ba as BaSO_4_ than dissolved Ba. Hypersaline untreated SG FPW that may spill or leak into surface water will pose a high toxicity risk to invertebrates in surface waters, particularly those more sensitive to salinity, such as *C. dubia*.

Organisms further downstream in open/flowing surface waters (stream or river) will be less affected by FPW due to greater dilutions and renewal of waters. However, it is expected that closed surface water systems, such as ponds, small dams, or low water flow streams that are not as easily renewed/refreshed or diluted, would be at an increased risk for the health of the biota within. The complex water chemistry of surface waters and FPW will influence the toxicity of dissolved Ba in addition to Na^+^ and Cl^-^. Consequently, single-species laboratory exposures like those in this study may underestimate population and community effects in an ecosystem.

## Supplementary Material

vgae019_Supplementary_Data

## Data Availability

Additional data is made available from the corresponding author, daniel.willems@student.rmit.edu.au

## References

[vgae019-B1] Alessi D. S. , Zolfaghari A., Kletke S., Gehman J., Allen D. M., Goss G. G. (2017). Comparative analysis of hydraulic fracturing wastewater practices in unconventional shale development: Water sourcing, treatment and disposal practices. Canadian Water Resources Journal / Revue Canadienne Des Ressources Hydriques, 42, 105–121. 10.1080/07011784.2016.1238782

[vgae019-B2] Arambašić M. B. , Bjelić S., Subakov G. (1995). Acute toxicity of heavy metals (copper, lead, zinc), phenol and sodium on *Allium cepa L*., *Lepidium sativum L*. and *Daphnia magna St*: Comparative investigations and the practical applications. Water Research, 29, 497–503. 10.1016/0043-1354(94)00178-A

[vgae019-B3] ASTM International (2014). Guide for conducting acute toxicity tests on test materials with fishes, macroinvertebrates, and amphibians. American Society for Testing and Materials International. 10.1520/E0729-96R14

[vgae019-B4] Atkinson M. J. , Bingman C. (1997). Elemental composition of commercial seasalts. Journal of Aquariculture and Aquatic Sciences, 8, 39–43.

[vgae019-B5] Australian and New Zealand Guidelines (2018). Australian and New Zealand guidelines for fresh and marine water quality. Australian and New Zealand Governments and Australian State and Territory Governments. https://www.waterquality.gov.au/anz-guidelines/guideline-values/default/water-quality-toxicants/search

[vgae019-B6] Aylward G. H. , Findlay T. J. V. (1974). SI chemical data (2nd ed) Wiley.

[vgae019-B7] Barton B. A. , Iwama G. K. (1991). Physiological changes in fish from stress in aquaculture with emphasis on the response and effects of corticosteroids. Annual Review of Fish Diseases, 1, 3–26. 10.1016/0959-8030(91)90019-G

[vgae019-B8] Bezirci G. , Akkas S. B., Rinke K., Yildirim F., Kalaylioglu Z., Severcan F., Beklioglu M. (2012). Impacts of salinity and fish-exuded kairomone on the survival and macromolecular profile of *Daphnia pulex*. Ecotoxicology (London, England), 21, 601–614. 10.1007/s10646-011-0820-0 22102012

[vgae019-B9] Bhoelan B. S. , Stevering C. H., van der Boog A. T. J., van der Heyden M. A. G. (2014). Barium toxicity and the role of the potassium inward rectifier current. Clinical Toxicology (Philadelphia, Pa.), 52, 584–593. 10.3109/15563650.2014.923903 24905573

[vgae019-B10] Biesinger K. E. , Christensen G. M. (1972). Effects of various metals on survival, growth, reproduction, and metabolism of *Daphnia magna*. Journal of the Fisheries Research Board of Canada, 29, 1691–1700. 10.1139/f72-269

[vgae019-B11] Birge W. J. , Black J. A., Westerman A. G., Short T. M., Taylor S. B., Bruser D. M., Wallingford E. D. (1985). Recommendations on numerical values for regulating iron and chloride concentrations for the purpose of protecting warm water species of aquatic life in the Commonwealth of Kentucky. Memorandum of Agreement, 5429.

[vgae019-B12] Blewett T. A. , Weinrauch A. M., Delompré P. L. M., Goss G. G. (2017). The effect of hydraulic flowback and produced water on gill morphology, oxidative stress and antioxidant response in rainbow trout (*Oncorhynchus mykiss*). Scientific Reports, 7, 46582. 10.1038/srep46582 28425455 PMC5397866

[vgae019-B13] Bonetti P. , Leuz C., Michelon G. (2021). Large-sample evidence on the impact of unconventional oil and gas development on surface waters. Science (New York, N.Y.), 373, 896–902. 10.1126/science.aaz2185 34413233

[vgae019-B14] Canadian Council of Ministers of the Environment (2011). *Canadian water quality guidelines for the protection of aquatic life: Chloride*. Canadian Council of Ministers of the Environment. https://ccme.ca/en/summary-table

[vgae019-B15] Cañedo-Argüelles M. , Hawkins C. P., Kefford B. J., Schäfer R. B., Dyack B. J., Brucet S., Buchwalter D., Dunlop J., Frör O., Lazorchak J., Coring E., Fernandez H. R., Goodfellow W., Achem A. L. G., Hatfield-Dodds S., Karimov B. K., Mensah P., Olson J. R., Piscart C., Timpano A. J. (2016). Saving freshwater from salts. Science (New York, N.Y.), 351, 914–916. 10.1126/science.aad3488 26917752

[vgae019-B16] Cañedo-Argüelles M. , Kefford B. J., Piscart C., Prat N., Schäfer R. B., Schulz C.-J. (2013). Salinisation of rivers: An urgent ecological issue. Environmental Pollution (Barking, Essex: 1987), 173, 157–167. 10.1016/j.envpol.2012.10.011 23202646

[vgae019-B17] Charles S. , Veber P., Delignette-Muller M. L. (2017). MOSAIC: A web-interface for statistical analyses in ecotoxicology. Environmental Science and Pollution Research International, 25, 11295–11302. 10.1007/s11356-017-9809-4 28842838

[vgae019-B18] Clausen M. V. , Hilbers F., Poulsen H. (2017). The structure and function of the Na, K-ATPase isoforms in health and disease. Frontiers in Physiology, 8, 371. 10.3389/fphys.2017.00371 28634454 PMC5459889

[vgae019-B19] Davies T. D. , Hall K. J. (2007). Importance of calcium in modifying the acute toxicity of sodium sulphate to *Hyalella azteca* and *Daphnia magna*. Environmental Toxicology and Chemistry, 26, 1243–1247. 10.1897/06-510R.1 17571691

[vgae019-B20] Dowden B. F. (1961). Cumulative toxicities of some inorganic salts to *Daphnia magna* as determined by median tolerance limits. Proc. A. Acad. Sci, 23, 77–85.

[vgae019-B21] Dowden B. F. , Bennett H. J. (1965). Toxicity of selected chemicals to certain animals. Journal - Water Pollution Control Federation, 37, 1308–1316. 5825886

[vgae019-B22] Dowse R. , Palmer C. G., Hills K., Torpy F., Kefford B. J. (2017). The mayfly nymph *Austrophlebioides pusillus* Harker defies common osmoregulatory assumptions. Royal Society Open Science, 4, 160520. 10.1098/rsos.160520 28280549 PMC5319315

[vgae019-B23] Elphick J. R. F. , Bergh K. D., Bailey H. C. (2011). Chronic toxicity of chloride to freshwater species: Effects of hardness and implications for water quality guidelines. Environmental Toxicology and Chemistry, 30, 239–246. 10.1002/etc365 20872898

[vgae019-B24] Elsner M. , Hoelzer K. (2016). Quantitative survey and structural classification of hydraulic fracturing chemicals reported in unconventional gas production. Environmental Science & Technology, 50, 3290–3314. 10.1021/acs.est.5b02818 26902161

[vgae019-B25] Erickson R. J. , Mount D. R., Highland T. L., Hockett J. R., Hoff D. J., Jenson C. T., Norberg-King T. J., Peterson K. N. (2017). The acute toxicity of major ion salts to *Ceriodaphnia dubia*. II. Empirical relationships in binary salt mixtures: Acute toxicity of binary mixtures of major ion salts to *C. dubia*. Environmental Toxicology and Chemistry, 36, 1525–1537. 10.1002/etc3669 27800634 PMC6157913

[vgae019-B26] Ferrer I. , Thurman E. M. (2015). Chemical constituents and analytical approaches for hydraulic fracturing waters. Trends in Environmental Analytical Chemistry, 5, 18–25. 10.1016/j.teac.2015.01.003

[vgae019-B27] Folkerts E. J. , Blewett T. A., Delompré P., Mehler W. T., Flynn S. L., Sun C., Zhang Y., Martin J. W., Alessi D. S., Goss G. G. (2019). Toxicity in aquatic model species exposed to a temporal series of three different flowback and produced water samples collected from a horizontal hydraulically fractured well. Ecotoxicology and Environmental Safety, 180, 600–609. 10.1016/j.ecoenv.2019.05.054 31132555

[vgae019-B28] Folkerts E. J. , Goss G. G., Blewett T. A. (2020). Investigating the potential toxicity of hydraulic fracturing flowback and produced water spills to aquatic animals in freshwater environments: A North American perspective. In de Voogt P. (Ed.), Reviews of environmental contamination and toxicology (Vol. 254, pp. 1–56). Springer International Publishing. 10.1007/398_2020_43 32318824

[vgae019-B29] Gardner K. M. , Royer T. V. (2010). Effect of road salt application on seasonal chloride concentrations and toxicity in south‐central Indiana streams. Journal of Environmental Quality, 39, 1036–1042. 10.2134/jeq2009.0402 20400599

[vgae019-B30] Golding L. A. , Kumar A., Adams M., Binet M., Gregg A., King J., McKnight K., Nidumolu B., Spadaro D., Kirby J. K. (2022). The influence of salinity on the chronic toxicity of shale gas flowback wastewater to freshwater organisms. Journal of Hazardous Materials, 428, 128219. 10.1016/j.jhazmat.2022.128219 35114525

[vgae019-B31] Golding L. A. , McKnight K., Binet M., Adams M., Apte S. C. (2018). Toxicity of dissolved and precipitated forms of barium to a freshwater alga (*Chlorella* sp. 12) and water flea (*Ceriodaphnia dubia*): Toxicity of dissolved and precipitated barium to aquatic biota. Environmental Toxicology and Chemistry, 37, 1632–1642. 10.1002/etc4107 29480964

[vgae019-B32] Goss G. , Alessi D., Allen D., Gehman J., Brisbois J., Kletke S., Zolfaghari A., Notte C., Thompson Y., Hong K. (2015). Unconventional wastewater management: A comparative review and analysis of hydraulic fracturing wastewater management practices across four North American basins. Canadian Water Network. 10.13140/RG.2.1.4812.1209

[vgae019-B33] Hall L. W. , Anderson R. D. (1995). The influence of salinity on the toxicity of various classes of chemicals to aquatic biota. Critical Reviews in Toxicology, 25, 281–346. 10.3109/10408449509021613 7576155

[vgae019-B34] Harmon S. M. , Specht W. L., Chandler G. T. (2003). A comparison of the daphnids *Ceriodaphnia dubia* and *Daphnia ambigua* for their utilization in routine toxicity testing in the southeastern United States. Archives of Environmental Contamination and Toxicology, 45, 79–85. 10.1007/s00244-002-0116-8 12948176

[vgae019-B35] Hoke R. A. , Gala W. R., Drake J. B., Giesy J. P., Flegler S. (1992). Bicarbonate as a potential confounding factor in cladoceran toxicity assessments of pore water from contaminated sediments. Canadian Journal of Fisheries and Aquatic Sciences, 49, 1633–1640. 10.1139/f92-182

[vgae019-B36] IBM Corp (2016). IBM SPSS Statistics for Mac OS (Version 24) [Computer software]. IBM Corp.

[vgae019-B37] Keating K. (1985). A system of defined (Sensu stricto) media for daphnid (Cladocera) culture. Water Research, 19, 73–78. 10.1016/0043-1354(85)90325-2

[vgae019-B38] Kefford B. J. , Hyne R. V., Brooks A. J., Bray J. P., Shenton M., Hills K., Nichols S. J. (2023). Single‐species acute lethal toxicity tests are not predictive of relative population and community effects of two salinity types. Limnology and Oceanography Letters, 8, 181–189. 10.1002/lol2.10208

[vgae019-B39] Khangarot B. S. , Ray P. K. (1989). Investigation of correlation between physicochemical properties of metals and their toxicity to the water flea *Daphnia magna Straus*. Ecotoxicology and Environmental Safety, 18, 109–120. 10.1016/0147-6513(89)90071-7 2806166

[vgae019-B40] Kondash A. J. , Albright E., Vengosh A. (2017). Quantity of flowback and produced waters from unconventional oil and gas exploration. The Science of the Total Environment, 574, 314–321. 10.1016/j.scitotenv.2016.09.069 27639468

[vgae019-B41] Kszos L. A. , Talmage S. S., Morris G. W., Konetsky B. K., Rottero T. (2003). Derivation of aquatic screening benchmarks for 1, 2-dibromoethane. Archives of Environmental Contamination and Toxicology, 45, 66–71. 10.1007/s00244-002-0151-5 12948174

[vgae019-B42] Kunz J. L. , Conley J. M., Buchwalter D. B., Norberg-King T. J., Kemble N. E., Wang N., Ingersoll C. G. (2013). Use of reconstituted waters to evaluate effects of elevated major ions associated with mountaintop coal mining on freshwater invertebrates: Use of reconstituted waters to evaluate effects of TDS. Environmental Toxicology and Chemistry, 32, 2826–2835. 10.1002/etc2391 24243594

[vgae019-B43] Lauer N. E. , Harkness J. S., Vengosh A. (2016). Brine spills associated with unconventional oil development in North Dakota. Environmental Science & Technology, 50, 5389–5397. 10.1021/acs.est.5b06349 27119384

[vgae019-B44] LeBlanc G. A. (1980). Acute toxicity of priority pollutants to water flea (*Daphnia magna*). Bulletin of Environmental Contamination and Toxicology, 24, 684–691. 10.1007/BF01608174 7459457

[vgae019-B45] Lilius H. , Hästbacka T., Isomaa B. (1995). *Short Communication*: A comparison of the toxicity of 30 reference chemicals to *Daphnia magna* and *Daphnia pulex*. Environmental Toxicology and Chemistry, 14, 2085–2088. 10.1002/etc5620141211

[vgae019-B46] MacLean M. M. , Doe K. G., Directorate C. E. P., Centre R. R. E. T. (1989). The comparative toxicity of crude and refined oils to Daphnia magna and Artemia. Environment Canada, Environmental Protection Directorate, River Road Environmental Technology Centre. https://books.google.com.au/books?id=EgtWGwAACAAJ

[vgae019-B47] Martínez-Jerónimo F. , Martínez-Jerónimo L. (2007). Chronic effect of NaCl salinity on a freshwater strain of *Daphnia magna Straus* (Crustacea: Cladocera): A demographic study. Ecotoxicology and Environmental Safety, 67, 411–416. 10.1016/j.ecoenv.2006.08.009 17055052

[vgae019-B48] McLaughlin M. C. , Borch T., McDevitt B., Warner N. R., Blotevogel J. (2020). Water quality assessment downstream of oil and gas produced water discharges intended for beneficial reuse in arid regions. The Science of the Total Environment, 713, 136607. 10.1016/j.scitotenv.2020.136607 31955100

[vgae019-B49] Meyer J. S. , Sanchez D. A., Bergman H. L., McWhorter D. B., Brookman J. A. (1985). Chemistry and aquatic toxicity of raw oil shale leachates from Piceance Basin, Colorado. Environmental Toxicology and Chemistry, 4, 559–572. 10.1002/etc5620040416

[vgae019-B50] Mount D. R. , Erickson R. J., Forsman B. B., Highland T. L., Hockett J. R., Hoff D. J., Jenson C. T., Norberg‐King T. J. (2019). Chronic toxicity of major ion salts and their mixtures to *Ceriodaphnia dubia*. Environmental Toxicology and Chemistry, 38, 769–783. 10.1002/etc4346 30569525 PMC6693352

[vgae019-B51] Mount D. R. , Erickson R. J., Highland T. L., Hockett J. R., Hoff D. J., Jenson C. T., Norberg-King T. J., Peterson K. N., Polaske Z. M., Wisniewski S. (2016). The acute toxicity of major ion salts to *Ceriodaphnia dubia*: I. influence of background water chemistry: Water chemistry effects on acute ion toxicity to *Ceriodaphnia*. Environmental Toxicology and Chemistry, 35, 3039–3057. 10.1002/etc3487 27167636 PMC6013840

[vgae019-B52] Mount D. R. , Gulley D. D. (1992). Development of a salinity/toxicity relationship to predict acute toxicity of saline waters to freshwater organisms. Gas Research Institute.

[vgae019-B53] Mount D. R. , Gulley D. D., Hockett J. R., Garrison T. D., Evans J. M. (1997). Statistical models to predict the toxicity of major ions to *Ceriodaphnia dubia, Daphnia magna* and *Pimephales promelas* (fathead minnows). Environmental Toxicology and Chemistry, 16, 2009–2019. 10.1002/etc5620161005

[vgae019-B54] National Industrial Chemicals Notification and Assessment Scheme (2017). *Identification of chemicals associated with coal seam gas extraction in Australia*. Project report prepared by the National Industrial Chemicals Notification and Assessment Scheme (NICNAS) as part of the National Assessment of Chemicals Associated with Coal Seam Gas Extraction in Australia. Commonwealth of Australia.

[vgae019-B55] Neff J. , Lee K., DeBlois E. M. (2011). Produced water: Overview of composition, fates, and effects. Produced Water, 3–54. 10.1007/978-1-4614-0046-2_1

[vgae019-B56] Neff J. M. (2008). Estimation of bioavailability of metals from drilling mud barite. Integrated Environmental Assessment and Management, 4, 184–193. 10.1897/IEAM_2007-037.1 17994916

[vgae019-B57] Ni Y. , Yao L., Sui J., Chen J., Liu F., Wang F., Zhu G., Vengosh A. (2022). Shale gas wastewater geochemistry and impact on the quality of surface water in Sichuan Basin. The Science of the Total Environment, 851, 158371. 10.1016/j.scitotenv.2022.158371 36041624

[vgae019-B58] Okamoto A. , Yamamuro M., Tatarazako N. (2015). Acute toxicity of 50 metals to *Daphnia magna*: Acute toxicity of 50 metals to *D. magna*. Journal of Applied Toxicology: JAT, 35, 824–830. 10.1002/jat.3078 25382633

[vgae019-B59] Oskarsson A. (2015). Barium. In Handbook on the toxicology of metals (pp. 625–634). Elsevier. 10.1016/B978-0-444-59453-2.00029-9

[vgae019-B60] Patterson L. A. , Konschnik K. E., Wiseman H., Fargione J., Maloney K. O., Kiesecker J., Nicot J.-P., Baruch-Mordo S., Entrekin S., Trainor A., Saiers J. E. (2017). Unconventional oil and gas spills: Risks, mitigation priorities, and state reporting requirements. Environmental Science & Technology, 51, 2563–2573. 10.1021/acs.est.6b05749 28220696

[vgae019-B61] Pérez-Fuentetaja A. , Goodberry F. (2016). *Daphnia’s* challenge: Survival and reproduction when calcium and food are limiting. Journal of Plankton Research, 38, 1379–1388. 10.1093/plankt/fbw077

[vgae019-B62] Pickering A. D. (1990). Stress and the suppression of somatic growth in teleost fish. Progress in Clinical and Biological Research, 342, 473–479. 2381949

[vgae019-B63] Renock D. , Landis J. D., Sharma M. (2016). Reductive weathering of black shale and release of barium during hydraulic fracturing. Applied Geochemistry, 65, 73–86. 10.1016/j.apgeochem.2015.11.001

[vgae019-B64] Robison A. L. (2011). Influence of predation-based chemical cues on contaminant sensitivity in fathead minnows (Pimephales promelas) and Daphnia pulex. Oklahoma State University.

[vgae019-B65] Schreck C. B. , Contreras-Sanchez W., Fitzpatrick M. S. (2001). Effects of stress on fish reproduction, gamete quality, and progeny. In Reproductive biotechnology in finfish aquaculture. (pp. 3–24). Elsevier. 10.1016/S0044-8486(01)00580-4

[vgae019-B66] Silva T. L. S. , Morales-Torres S., Castro-Silva S., Figueiredo J. L., Silva A. M. T. (2017). An overview on exploration and environmental impact of unconventional gas sources and treatment options for produced water. Journal of Environmental Management, 200, 511–529. 10.1016/j.jenvman.2017.06.002 28628868

[vgae019-B67] Soucek D. J. , Linton T. K., Tarr C. D., Dickinson A., Wickramanayake N., Delos C. G., Cruz L. A. (2011). Influence of water hardness and sulfate on the acute toxicity of chloride to sensitive freshwater invertebrates. Environmental Toxicology and Chemistry, 30, 930–938. 10.1002/etc454 21191883

[vgae019-B68] Stringfellow W. T. , Domen J. K., Camarillo M. K., Sandelin W. L., Borglin S. (2014). Physical, chemical, and biological characteristics of compounds used in hydraulic fracturing. Journal of Hazardous Materials, 275, 37–54. 10.1016/j.jhazmat.2014.04.040 24853136

[vgae019-B69] Tan Q.-G. , Wang W.-X. (2010). Interspecies differences in calcium content and requirement in four freshwater cladocerans explained by biokinetic parameters. Limnology and Oceanography, 55, 1426–1434. 10.4319/lo.2010.55.3.1426

[vgae019-B70] United States Environmental Protection Agency (1988). Ambient water quality criteria for chloride—1988. United States Environmental Protection Agency. https://www.epa.gov/sites/default/files/2018-08/documents/chloride-aquatic-life-criteria-1988.pdf

[vgae019-B71] United States Environmental Protection Agency (2002a). Methods for measuring the acute toxicity of effluents and receiving waters to freshwater and marine organisms. (Report No EPA-821-R-02–012). United States Environmental Protection Agency.

[vgae019-B72] United States Environmental Protection Agency (2002b). Short-term methods for estimating the chronic toxicity of effluents and receiving waters to freshwater organisms (Report No. EPA-821-R-02-013). United States Environmental Protection Agency.

[vgae019-B73] United States Environmental Protection Agency (2010). National pollutant discharge elimination system (NPDES) permit writers’ manual (Report No. EPA-833-K-10-001). United States Environmental Protection Agency. https://www.epa.gov/sites/default/files/2015-09/documents/pwm_2010.pdf

[vgae019-B74] United States Environmental Protection Agency (2023). ECOTOX knowledgebase. United States Environmental Protection Agency. https://cfpub.epa.gov/ecotox/index.cfm

[vgae019-B75] Valenti T. W. , Cherry D. S., Neves R. J., Locke B. A., Schmerfeld J. J. (2007). Case study: Sensitivity of mussel glochidia and regulatory test organisms to mercury and a reference toxicant. In J. L. Farris & J. H Van Hassel (Eds.), Freshwater bivalve ecotoxicology. (pp. 351–367). CRC Press.

[vgae019-B76] Verbruggen E. M. J. , Smit C. E., Van Vlaardingen P. L. A. (2020). Environmental quality standards for barium in surface water: Proposal for an update according to the methodology of the Water Framework Directive. 10.21945/RIVM-2020-0024

[vgae019-B77] Warner N. R. , Christie C. A., Jackson R. B., Vengosh A. (2013). Impacts of shale gas wastewater disposal on water quality in western Pennsylvania. Environmental Science & Technology, 47, 11849–11857. 10.1021/es402165b 24087919

[vgae019-B78] Waxman H. A. , Markey E. J., DeGette D. (2011). Chemicals used in hydraulic fracturing. *United States House of Representatives Committee on Energy and Commerce Minority Staff*. http://www.damascuscitizensforsustainability.org/wp-content/uploads/2012/03/dems.energy.Hydraulic-Fracturing-Report-4.18.11.pdf

[vgae019-B79] Willems D. J. , Kumar A., Nugegoda D. (2022). The acute toxicity of salinity in onshore unconventional gas waters to freshwater invertebrates in receiving environments: A systematic review. Environmental Toxicology and Chemistry, 41, 2928–2949. 10.1002/etc5492 36193756 PMC9828407

[vgae019-B80] Willems D. J. , Kumar A., Nugegoda D. (2023). Mixture toxicity of three unconventional gas fracking chemicals, barium, o‐cresol, and sodium chloride, to the freshwater shrimp *Paratya australiensis*. Environmental Toxicology and Chemistry, 42, 481–494. 10.1002/etc5538 36511521 PMC10107621

