# Supplementary material for: Chronic toxicity of dissolved barium and sodium chloride to the water flea Ceriodaphnia dubia: implications for unconventional gas flowback–produced waters
Source: Environ Toxicol Chem. 2025 Jan 6;44(1):169–83. doi: 10.1093/etojnl/vgae019 (PMC11790209; doi:10.1093/etojnl/vgae019)
Supplement: vgae019_Supplementary_Data [file vgae019_supplementary_data.zip › vgae019_Supplementary_Data/Chronic toxicity... - SUPP. FIGURES (ETCJ-Jun-24-00349).docx]

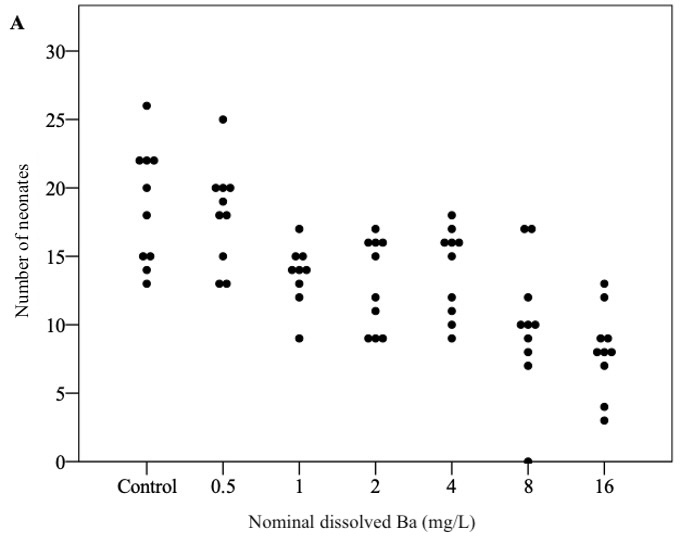


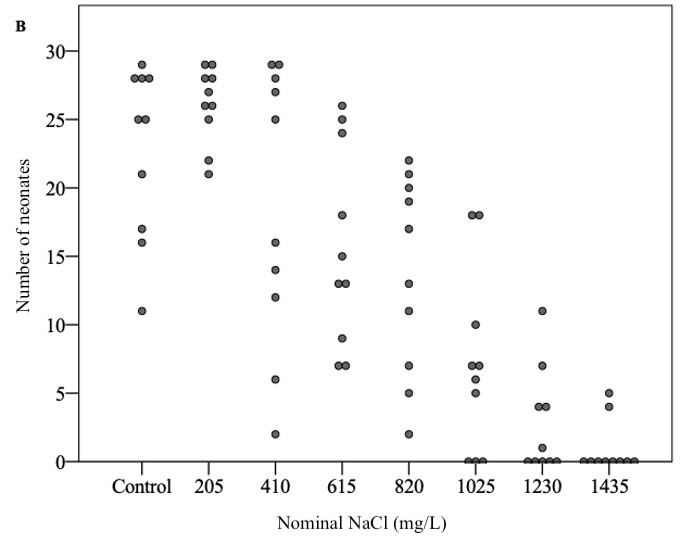


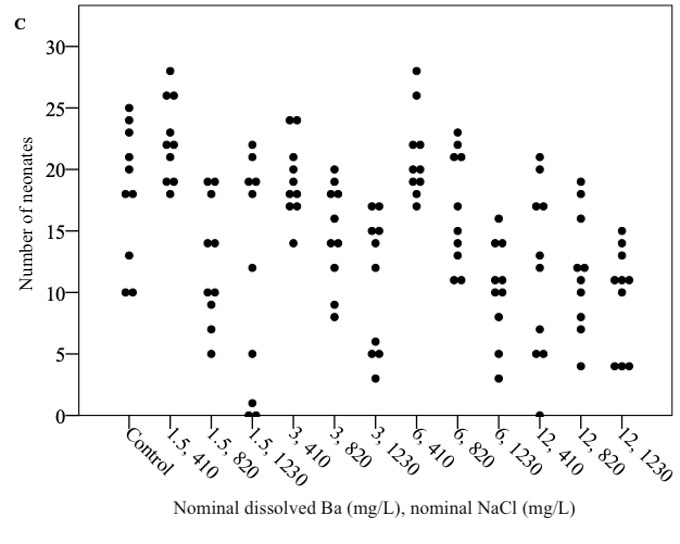


**Figure S1:** Total number of neonates reproduced for each parent *Ceriodaphnia dubia* by treatments from the three chronic exposures to **A**) 7-day dissolved Ba (mg/L), **B)** 6-day NaCl (mg/L), **C)** 7-day binary dissolved Ba (mg/L) and NaCl (mg/L). For all unique pairwise combinations (Tukey’s post hoc) of significance between groups, see Table S3. Plots were generated based on the reproductive data within Tables S6-S8.

Footnote:

NaCl is at concentrations additional to the control as Na^+^ and Cl^-^ ions are present in moderately hard water (MHW) salts.
